# Young Stroke Mortality in Fiji Islands: An Economic Analysis of National Human Capital Resource Loss

**DOI:** 10.5402/2012/802785

**Published:** 2012-06-21

**Authors:** Jagdish C. Maharaj, Mahendra Reddy

**Affiliations:** ^1^Lourdes Hospital and Community Health Service, P.O. Box 974, Dubbo, NSW 2830, Australia; ^2^College of Business, Hospitality and Tourism Studies, Fiji National University, P.O. Box 7222, Nasinu, Fiji

## Abstract

*Introduction*. The objective of this study was to perform an economic analysis in terms of annual national human capital resource loss from young stroke mortality in Fiji. The official retirement age is 55 years in Fiji. *Method*. Stroke mortality data, for working-age group 15–55 years, obtained from the Ministry of Health and per capita national income figure for the same year was utilised to calculate the total output loss for the economy. The formula of output loss from the economy was used. *Results*. There were 273 stroke deaths of which 53.8% were of working-age group. The annual national human capital loss from stroke mortality for Fiji for the year was calculated to be F$8.85 million (US$5.31 million). The highest percentage loss from stroke mortality was from persons in their forties; that is, they still had more then 10 years to retirement. *Discussion*. This loss equates to one percent of national government revenue and 9.7% of Ministry of Health budget for the same year. The annual national human capital loss from stroke mortality is an important dimension in the overall economic equation of total economic burden of stroke. *Conclusion*. This study demonstrates a high economic burden for Fiji from stroke mortality of young adults in terms of annual national human capital loss.

## 1. Introduction

*“Stroke is a cause of poverty and is caused by poverty” *[1]*.*

The epidemiological changes through advances in socioeconomic developments and changing demographics have altered the profile of the major causes of mortality and morbidity. Fiji is undergoing transition as is occurring in most other developing regions of the world. Rapid urbanization and globalisation have brought changes in life styles that have produced a surge in mortality and morbidity noncommunicable diseases (NCDs) such as cancer, cardiovascular diseases including stroke and ischaemic heart disease, chronic obstructive pulmonary diseases, and mental disorders. NCDs account for 62% of all deaths and 50% of the disability burden in the Economic and Social Commission for Asia and the Pacific region [2] and are at the point of a global crisis [3]. Several reports in the literature implicate stroke as the third leading cause of death and an important cause of hospital admission and long-term disability [4–6]. Over the decades some decline in stroke mortality has also been reported [7–9]. 

Stroke incidence ranges between 7 and 15 per 100,000 people/year [10] to 8.63 to 19.12 per 100,000 people/year [11] with substantial global variation in the relative burden of stroke [12]. Increasing burden of stroke, particularly in low-to-middle income countries like Fiji has been reported. Feigin et al. in a review showed a divergent, statistically significant trend in stroke incidence rates over the past four decades, with a 42% decrease in stroke incidence in high-income countries and a greater than 100% increase in stroke incidence in low-to-middle income countries [13]. The World Health Organisation (WHO) in its NCD Surveillance: STEPwise approach to stroke surveillance estimated that in 2002 strokes resulted in 5.5 million deaths worldwide, making it the leading cause of death, and the projections to the year 2020 indicate that the number of people suffering from stroke each year will substantially increase, the majority of the disease burden being in the low-to-middle income countries [14]. Stroke in young not only poses different set of challenges for the individual, family, and society requiring different approach to investigation and management [10] due to differing etiological nature compared to the aged population, but the affection of economically productive person in prime of their life, particularly in developing countries, adds further to the disease burden [15]. Stroke case fatality in 18–40-year age group has been reported as 4.2% and 19.7% at 24 and 72 hours, respectively [16].

## 2. Fiji and Noncommunicable Diseases

Fiji, a multiethnic, upper middle income [17] country with GDP of US$4,600 and a population of 837,271 [18] lies in the South Western Pacific region. Stroke and ischaemic heart disease are the leading causes of premature disability and death [19]. Over 50% of deaths were attributed to NCDs in a survey in 1980 which rose to 82% in 2000 with stroke and coronary heart disease accounting for just over 30% of all deaths in the 40–59-year age group [20]. WHO had projected that if the current world trends in NCDs continue, they would, by 2020, probably account for 73% of deaths and 60% of the total world disease burden. This had already been surpassed in Fiji in 2004 [21], that is, nineteen years earlier then the WHO prediction! Recent report indicates 82% deaths in Fiji attributed to NCDs [22] with the economic impact beyond the costs to health services and increased governmental financial allocation for prevention programs [23]. There is high prevalence of chronic disability in Fiji, largely preventable diseases in persons of working age and among the young old (60–74 years) [24], a rising incidence of stroke [25], a high prevalence of comorbidities [26], and the trend towards an earlier onset of disability evident in Fiji's population [27]. The average age at which people develop diabetes and cardiovascular disease is getting lower. In Fiji,due to premature deaths only 16% of the population is aged more than 55 years [28]. The projections suggest that the incidence of stroke will rapidly increase in the near future [29]. The reported top ten stroke risk factors are probably very similar in Fiji [30]. The provision of acute care and rehabilitative services for stroke survivors needs enhancement [31].

## 3. Measures of Economic Burden of Stroke: DALYs and Dollars

Burden of stroke studies discusses cost of resource utilisation, loss of quality of life, or loss of life (mortality). Stroke imposes substantial economic burden at both microeconomic (individual/household) and macroeconomic (societal) levels. Some 24% to 33% stroke survivors report economic difficulties with return to work ranging from 0% to 100% [32]. In Fiji, at the microeconomic level when the “only breadwinner” in the family has had a stroke the loss of financial support for the family can be devastating. Kepa and Makutu [33] noted that “premature deaths in Fiji is a great concern with the implications of poverty created from loved ones lost to noncommunicable diseases becoming a serious threat.” There are numerous studies from various countries addressing economic cost of resources utilisation following stroke [34–42] including economic evaluation recommendation of identification, measurement, and valuation, and then comparison of the costs (inputs) and benefits (outcomes) of two or more alternative treatments or activities [43, 44]. A review reported the average costs of stroke ranged from US$468 to US$146,146 [45].

A measure for quality of life presented as estimated quality-adjusted life years (QALYs) saved or disability-adjusted life years (DALYs) [46]. One DALY is one year of “healthy” life lost, and the burden of disease is a measurement of the gap between the current health of the population and an ideal situation in which everyone in the population lives to old age in full health [47]. In Australia, the first burden of disease and injury study using DALYs carried out using World Bank and WHO Global Burden of Disease Study methodology revealed that stroke and ischaemic heart disease were the leading causes of the total disease burden together accounting for nearly 18% of the total disease burden [19]. The available literature on the economic analysis of stroke largely deals with economic cost of care and/or resource utilisation following stroke or is expressed as disease burden as disability-adjusted life years (DALYs). The total economic burden of stroke in Fiji would include cost of resource utilisation, direct health care costs, indirect health and other costs, intangible costs, cost of carer, carers' productivity losses, and stroke survivors' productivity losses due to stroke morbidity and mortality. 

## 4. Human Capital

Human capital is main value of modern society and basic factor of economic achievements and refers to the stock of skills and knowledge embodied in the ability to perform labour so as to produce economic value. Such stock valuable know-how embodied in the labour force can be seen as surprisingly analogous to physical capital such as machines and buildings [48]. Human capital typically requires investment to be created in form of education and training, it has a market value, and it can depreciate with time [48]. In a society which places a strong emphasis on competition, financial return and viability, the “people issues” can sometimes be neglected. The theory of the human capital has appeared as a result of the application of principles of the economic theory to problems of economy of public health services, derivation, bionomics, and migration [49]. Stockley described the term human capital as recognition that people in organisations and businesses are important and essential assets who contribute to development and growth, in a similar way as physical assets such as machines and money, and that the collective attitudes, skills, and abilities of people contribute to national performance and productivity [50]. 

## 5. Method

This study performed an economic analysis of stroke mortality in terms of annual national human capital resource loss from premature deaths of young working age group in Fiji. The official retirement age is 55 years in Fiji. The loss from stroke mortality was calculated using the annual stroke mortality data obtained from Fiji Ministry of Health [51]. In Fiji stroke diagnosis is largely clinical, and the Ministry of Health mortality data used in this study is based on the clinicians' record on the death certificates. All stroke mortality aged 15–55 years was included in the analysis. An estimation of economic burden of stroke in terms of national human capital loss with some degree of accuracy can be made. There were 273 stroke deaths during the year of which 147 met the inclusion criteria being between ages of 15–55 years. 

The costs of human capital resource loss may include income foregone following stroke, cost of training, recruitment and replacement of labour force, increased medical and health care costs, costs of recruiting carer(s), and loss of family carers' financial income. The loss to the economy arising from stroke of a productive worker is given by the discounted value of the output foregone over the period in which the worker is not able to earn an income, which will include period starting immediately from the onset of stroke to return to work or death. A discount rate of eight percent (8%) was utilized to compute the present value. The maximum replacement period could be the remaining working lifespan, which is estimated by subtracting the age of the individual at stroke from the retirement age. The annual income foregone multiplied by the number of work years lost will yield the total income foregone which must be discounted using a suitable discount rate. This, of course, assumes that the individual's annual income remains the same throughout the remaining working period.

Due to varying levels of income for individuals, the average annual income is proxied by the per-capita income. The present discounted value is the value of money today (in the present) of a payment that is or was promised to be made in the future or in the past. A formula to compute the present discounted value (PV) of the income foregone is as follows:

(1)Output  Loss  from  the  Economy=S(1r)[1−1(1+r)t],

where, *S* is the annual income foregone, *r* is the chosen rate of discount, and *t* is the working lifespan lost as the result of stroke. 

The friction-cost method has been put forward as an alternative to the human-capital method as it allows more realistic estimates of productivity costs to be calculated for use in economic evaluations. The possibility of replacement of absentees is at the heart of the friction-cost method. It recognises that society will restore initial production levels after some period of adaptation, the length of which may depend on the availability of labour and, hence, on unemployment rate, which was 7.6% for Fiji. The friction-cost method has received two main criticisms in the literature: (i) it has no theoretical underpinning, and (ii) it treats leisure time as having no value. Brouwer and Koopmanschap [52] demonstrated in a “theoretical” time-allocation model how time use shifts in the friction-cost method, and that leisure is not treated as having no value. Rather, it is considered to be valued in terms of QALYs as is normally the case in economic evaluation. The time-allocation model also demonstrates that when using the friction-cost or human-capital method the changes in the amount of unpaid work and leisure time need to be valued separately. These changes should be incorporated into economic analyses. 

Another approach, which can be applied to evaluate resource utilization with ranking option of intervention, is the model of resource utilization, costs, and outcome for stroke (MORUCOS) [53]. In the trial with the application of this methodology aspirin, a low-cost intervention applicable to a large number of stroke patients was evaluated against recombinant tissue-type plasminogen activator (rtPA). Analysis of health benefits, in terms of dollars and DALYs, could be produced, and the authors concluded that, if used to assess interventions across the stroke care continuum, MORUCOS offers enormous capacity to support decision making in the prioritising of stroke services. Further validation of the methodology suggests that MORUCOS is transparent and flexible in describing Australian stroke care and can effectively be used to systematically evaluate a range of different interventions even adjusting to account for stroke subtypes [54].

According to the WHO comprehensive guide to identifying the economic consequences of disease, and injury document this analysis is *“Full-income models”* approach which estimates value of statistical life (VSL) to years lost to disease which goes beyond purely market-based losses and as stated represents only partial estimates [55]. In this study, we use the human capital loss method as opposed to “Frictional Cost” method. In the frictional cost method, it is assumed that the worker is replaced at a later date. However, with the stroke victims' death, the worker is taken to be totally out of the economic system. Thus, the Human Capital Loss method is more appropriate. This study utilized the formula, discussed above, to compute the present value (PV) of the income foregone from stroke mortality in Fiji.

## 6. Result

Utilizing the latest available stroke mortality data obtained from Fiji Ministry of Health and a per capita National Income figure of F$5,131.50 (US$3,078.90) for the same year, with a discounted rate of 8%, the total output loss for the economy was calculated. There were 147 young stroke deaths of working-age group comprising 53.8% of all stroke mortality. As presented in Table 1, the annual national human capital loss from stroke mortality of young working-age persons for Fiji was calculated to be F$8.85 million (US$5.31 million). 

**Figure 1:**
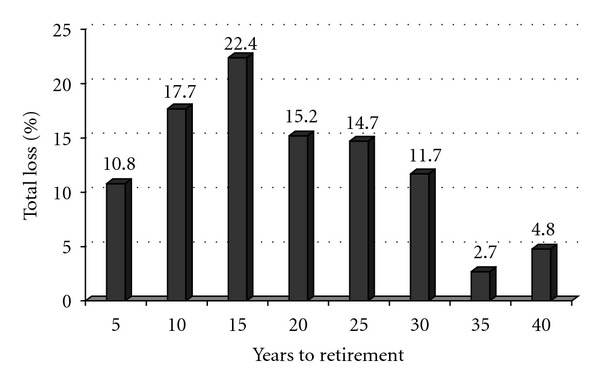
Years to retirement and percentage of total output loss from stroke mortality.

Amongst the stroke deaths 50% were 1–14 years and the other 50% 15–40 years to retirement age.  Figure 1 shows that the highest percentage loss from stroke mortality was from persons in their early and midforties; that is, they still had more then 10 years to retirement age of 55 years in Fiji.

## 7. Discussion

The calculated national human capital resource loss from young stroke mortality for Fiji of F$8.85 million (US$5.31 million) is comparatively one percent of the national government revenue of F$895.99 million (US$537.59 million) [56] and almost ten percent (9.7%) of the Ministry of Health's total budget of $91.02 million (US$54.61 million) [57] for the year. In this context, it is a substantial loss to Fiji's economy. Although it is recognised that productivity losses may begin immediately following stroke, this paper specifically and only addressed the issue of human capital loss following young stroke mortality of working-age young adults. It is important to note that the loss is a direct function of the number of working-age people who died from stroke. Therefore, if there is an increase in the incidence of stroke and mortality, this figure will also increase, thus raising the national economic loss.

This national human capital loss calculation used 55 years as the retirement age in Fiji. However, many people may remain gainfully employed past the age of 55 years in formal paid employment or informal unpaid work, caring and supporting their families [58]. Stroke incidence (and associated mortality) in Fiji has been projected to rapidly increase with increasing age [29, 59]. Thus, if stroke mortality for ages above 55 years of age for those making an economic contribution at both micro- and macroeconomic levels is factored into the equation, the actual national human capital loss could be much higher from stroke mortality in Fiji.

In 2002, the estimated DALY's losts to stroke in Fiji was 1,442 which represented 7.1% of the total loss (20,234 DALY's) from all causes in Fiji [60]. Tenfold difference in rates of stroke mortality and DALY loss between the most-affected and the least-affected countries has been reported with rates of stroke mortality and DALY loss being highest in eastern Europe, north Asia, central Africa, and the south Pacific [61]. The national per capita income, used in this study for the calculation of the national human capital resource loss happens to be the strongest predictor of mortality and DALY loss, rates (*P* < 0.0001) even after adjustment for cardiovascular risk factors (*P* < 0.0001) [61].

Apart from primary prevention of stroke, it is also very important to provide good acute medical care to prevent complications and mortality from stroke as well as providing adequate rehabilitative measures to return capable stroke survivors to productive living in the community. Improved acute care and rehabilitation will reduce the economic burden of stroke—particularly which relates to medical complications and premature mortality. It is evident that admission to hospital [62], organised acute stroke care [63–65] preferably in a specialist multidisciplinary stroke unit has better outcome both in terms of immediate survival and long-term functional outcome after rehabilitation.

There was no similar study on national human capital resource loss from stroke mortality found in the literature. However, Evers et al. [66] conducted a literature search from January 1966 to July 2003. They systematically reviewed 25 stroke cost studies and reported that the proportion of national health care budget for stroke in the eight countries studied was unequivocal for the more recent studies and was approximately 3% of total health care expenditures. In this context, Fiji's loss of an equivalent of 9.7% of health care budget due to young stroke mortality alone, not taking into account the “health care expenditures on stroke,” is comparatively much higher than the reported 3%.

This study specifically evaluated one aspect of economic burden of stroke in Fiji. A comprehensive study of economic burden of stroke, including resource utilisation, direct health care costs, indirect carer costs, intangible costs, carers productivity losses, and stroke survivors productivity losses due to stroke morbidity, was beyond the scope of this study. To calculate stroke survivors productivity losses due to stroke morbidity one needs to have data and take into account as to how long a stroke survivor gave up employment as a result of their stroke and whether the stroke survivors returned to productive work, at what level and for how long after the stroke.

## 8. Conclusion

Although this study provides only one important aspect of the economic burden of stroke in Fiji, it demonstrates that the economic loss from young working-age stroke mortality can be substantial and is not well reported in literature. This methodology can be utilized to give a fairly accurate view of economic burden not only from stroke mortality but other causes of premature mortality such as coronary artery disease, trauma, and other causes. The economic burden from premature deaths in Fiji has been raised as a growing concern to families and health workers [33]. There is lack of data on economic burden of stroke in Fiji; thus, there is opportunity for further research to evaluate many different aspects of economic burden of stroke in Fiji.

## Figures and Tables

**Table 1:** Net present value of output loss from stroke mortality.

Number died	Years to retirement	Discounted value of output loss per person		Total loss	
		F$000	US$000	F$000	US$000

3	40	140.4	84.2	421.2	252.7
2	35	117.7	70.6	235.4	141.2
9	30	114.9	68.9	1,034.1	620.5
13	25	100.2	60.1	1,302.6	781.6
16	20	83.9	50.3	1,342.4	805.4
30	15	65.9	39.5	1,977.0	1186.2
34	10	46.1	27.7	1,567.4	940.4
40	5	24.2	14.5	968.0	580.8

Total				8,848.1	5,308.9
